# A Bayesian Model for Pooling Gene Expression Studies That Incorporates Co-Regulation Information

**DOI:** 10.1371/journal.pone.0052137

**Published:** 2012-12-28

**Authors:** Erin M. Conlon, Bradley L. Postier, Barbara A. Methé, Kelly P. Nevin, Derek R. Lovley

**Affiliations:** 1 Department of Mathematics and Statistics, University of Massachusetts, Amherst, Massachusetts, United States of America; 2 Department of Microbiology, University of Massachusetts, Amherst, Massachusetts, United States of America; University of Georgia, United States of America

## Abstract

Current Bayesian microarray models that pool multiple studies assume gene expression is independent of other genes. However, in prokaryotic organisms, genes are arranged in units that are co-regulated (called operons). Here, we introduce a new Bayesian model for pooling gene expression studies that incorporates operon information into the model. Our Bayesian model borrows information from other genes within the same operon to improve estimation of gene expression. The model produces the gene-specific posterior probability of differential expression, which is the basis for inference. We found in simulations and in biological studies that incorporating co-regulation information improves upon the independence model. We assume that each study contains two experimental conditions: a treatment and control. We note that there exist environmental conditions for which genes that are supposed to be transcribed together lose their operon structure, and that our model is best carried out for known operon structures.

## Introduction

The wealth of gene expression data currently being produced has created an urgent need for new statistical methods to analyze and pool this information. A common goal of gene expression studies is to identify genes that are differentially expressed between two conditions, such as wildtype versus mutant or treatment versus control. Bayesian and empirical Bayesian models have been developed extensively for individual microarray studies (Baldi and Long [1]; Efron et al. [2]; Newton et al. [3], [4]; Tseng et al. [5]; Broët et al. [6]; Ibrahim et al. [7]; Lönnstedt and Speed [8]; Townsend and Hartl [9]; Gottardo et al. [10]; Ishwaran and Rao [11], [12]; Kendziorski et al. [13]; Do et al. [14]; Lönnstedt and Britton [15]), and several Bayesian approaches have recently been introduced to combine multiple microarray studies (Choi et al. [16]; Shen et al. [17]; Jung et al. [18]; Conlon et al. [19], [20]; Scharpf et al. [21]; see also Tseng et al. [22] for a comprehensive review of meta-analysis methods). Choi et al. [16] introduced the first Bayesian meta-analysis model to detect differentially expressed genes between two experimental conditions. This approach combined standardized gene effects into an overall mean effect across studies, and included an inter-study variability parameter in the model. Shen et al. [17] implemented a Bayesian model within each separate study to transform gene expression measures to expression probabilities. The converted data was pooled across studies to identify prognostic markers for disease. In this method, Bayesian models were used for data pre-processing, but not as a data integration procedure. Jung et al. [18] introduced a Bayesian model-based clustering method for meta-analysis to identify differentially-expressed genes between two samples. This model specified a normal mixture prior distribution for the gene effects, with the number of components unknown. The number of components was calculated by first modeling a large number, e.g. 10, and counting the number of non-empty components in the observed results. Similar to Choi et al. [17], Jung et al. [18] pooled standardized gene effect size estimates into an overall mean effect across studies, and included a parameter of inter-study variability in the model. Unlike these previous methods, Conlon et al. ([19], [20]) introduced a Bayesian meta-analysis model that treated each study separately, combining only probabilities of differential expression without integrating expression values. In a comparative study of Bayesian meta-analysis models, Conlon et al. [20] found that combining only probabilities of differential expression outperformed pooling expression measures across studies, for their data sets.

The current Bayesian meta-analysis models assume that the average expression of a gene is independent of other genes. However, in prokaryotic species, many genes are organized in operons, which consist of two or more genes that are next to each other on the chromosome and commonly transcribed. Genes within an operon tend to have similar levels of expression (Xiao et al. [23]); this fact is commonly used in predicting operon structure (Sabatti et al. [24]; Bockhorst et al. [25]). More specifically, Xiao et al. [23] examined 217 microarray experiments for 53 conditions of the bacterium *Escherichia coli*. They found high correlation of expression among pairs of genes in predicted operons (mean correlation 0.62), and correlation near zero for randomly selected pairs of genes (mean correlation 0.012). Based on these findings, Xiao et al. [23] developed a Bayesian model for individual microarray studies that incorporated predicted operon structure; this model borrowed information across genes within an operon to estimate gene expression levels. The authors found that incorporating operon structure into the model improved the detection of differentially expressed genes versus an independence model for one study. Additional Bayesian models for individual microarray studies have included operon structure as prior information in the models (Price et al. [26]; Pin et al. [27]). However, operon structure has not previously been incorporated into Bayesian meta-analysis models for microarray data. Here, we develop a new Bayesian meta-analysis model that incorporates operon information into the model. Our Bayesian meta-analysis operon model borrows information from genes within the same operon; our model then produces the posterior probability of differential expression for each gene. This posterior probability of differential expression is the basis for inference. We found in simulations of two and five studies that our operon model outperformed the independence model by using three comparison measures: the proportion of true genes discovered in meta-analysis versus individual studies, the number of true genes discovered for fixed levels of Bayesian false discovery, and the number of true discoveries for a fixed top number of genes. When pooling two *Geobacter* (*G.*) *sulfurreducens* microarray studies, we show that the operon model produces higher proportions of discovered genes in meta-analysis versus separate analyses than the independence model. In addition, for the same thresholds of Bayesian false discovery, we illustrate that the operon model identifies more discoveries than the independence model for this biological data. We note that there exist environmental conditions for which genes that are supposed to be transcribed together lose their operon structure, and that our model is best carried out for known operon structures.

## Methods

### Bayesian Meta-analysis Independence Model

Biologists frequently carry out independent microarray studies for the same biological system or pathway; often using different technologies. For example, Methé et al. [28] used spotted DNA microarrays to examine nitrogen fixation in *G. sulfurreducens*. Alternatively, Postier et al. [29] studied this same pathway using CombiMatrix short oligonucleotide arrays (for further details of the biological data, see Appendix S1: Biological data). By combining the two studies, we increase the sample size and more precisely identify true target genes. More broadly, data typically consists of multiple independent studies for one biological system, with two conditions: a treatment and control; Bayesian meta-analysis models integrate this information in a systematic way. The following model combines studies from two different platforms, spotted and oligonucleotide arrays, and assumes that the average expression of a gene is independent of other genes. It is similar to the model introduced by Conlon et al. [19]; the spotted array study consists of replicate slides within repeated experiments, and the oligonucleotide array study contains multiple probes, slides and experiments. We specify Model (1) as follows.

For spotted array (SA) studies:
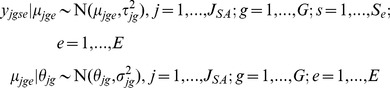



For oligonucleotide array studies:
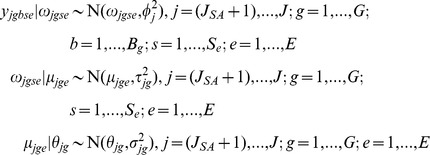



For all studies:
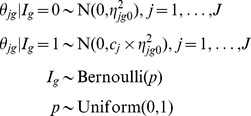
(1)


For the spotted array studies, the *y_jgse_* are the observed data, and are the normalized log-expression ratios for study *j*, gene *g*, slide *s*, and experiment *e*. These are the log-ratios of fluorescent intensity levels for the mRNA of the control and treatment samples, which are labelled green and red (Cy3 and Cy5). The *y_jgse_* values are standardized so that each slide had zero mean and unit standard deviation (see also Shen et al. [17]; Conlon et al. [19], [20]). This model takes into account that the *y_jgse_* are influenced by slide and experiment variance. Within each study, *y_jgse_* is modeled as a sample from a normal distribution of gene-specific slide values within an experiment, denoted as 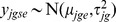. Here *µ_jge_* is the gene-specific average of all slide values in an experiment, and 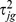 represents the slide variability. In turn, the within-experiment mean *µ_jge_* is modeled as a sample from a normal distribution of experiment values, denoted as 
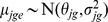
. Here, *θ_jg_* is the average log-expression ratio of gene *g* for study *j,* and 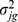 indicates the experiment variance.

For the oligonucleotide microarrays, termed *in-situ* synthesized oligonucleotide (ISO) arrays, each gene is characterized by up to four probes on each array (further detail is provided in Appendix S1: Biological data). For Model (1), the *y_jgbse_* are the normalized log-ratios of expression for study *j*, gene *g*, probe *b*, slide *s*, and experiment *e*. These are again the ratio of fluorescent intensity levels for the treatment and control mRNA samples, labelled red and green (Cy5 and Cy3), standardized so that each slide had zero mean and unit standard deviation. Here, the *y_jgbse_* are influenced by the probe, slide and experiment variance. For each study, the *y_jgbse_* are modeled as gene-specific samplings from normal distributions of probe values within each slide. This is denoted as 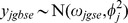, where *ω_jgse_* is the mean among all probe values for a slide for each gene, and 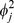 represents the variability across probes. A common probe variance 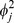 is assumed; this value is calculated from the data, similar to other approaches (e.g. Xiao et al. [23]). The within-slide mean *ω_jgse_* denotes a sampling from a normal distribution of slide values; this is modeled as 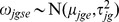. Here, *µ_jge_* is again the gene-specific average for all slide values of an experiment, and 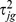 again measures the slide variability. The remaining parameters are as described previously for spotted arrays.

The *θ_jg_* values are modeled as a normal distribution with mean zero and small variance for non-expressed genes, and with large variance for differentially expressed genes. Note that Model (1) specifies each study individually, and does not pool the mean expression values for each study into an overall mean. In addition, only the *y_jgse_* and *y_jgbse_* values are observed; the remaining model parameters are unobserved.

We define *I_g_* ∼ Bernoulli(*p*) as the gene-specific indicator variable for differential expression, i.e. *θ_jg_* ≠ 0, *j* = 1,…, *J*, where *p* is the percent of differentially expressed genes. Thus, Prob(*I_g_* = 1) = *p*, where
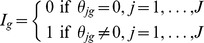



Here, genes are separated into two groups, non-expressed (*I_g = _*0) and differentially expressed (*I_g = _*1) with probabilities (1-*p*) and *p*, respectively. When *I_g = _*0, the *θ_jg_* are modeled as normally distributed around zero with small variance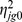; when *I_g = _*1, the *θ_jg_* are modeled as normally distributed around zero with large variance 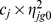. Model (1) produces the gene-specific posterior probability of differential expression, *D_g_* = Prob(*I_g = _*1 | data), which is used for inference.

### Bayesian Meta-analysis Operon Model

The previous Model (1) assumed that the average expression for a gene is independent of other genes. However, in prokaryotic genomes, many genes are organized in operons, which are commonly transcribed. Thus, genes in the same operon tend to have similar expression levels. Here, we introduce a new Bayesian meta-analysis model that incorporates predicted operon structure into the model. Our model borrows information across operons, and used a weighted average of the individual gene’s expression level and the operon expression level to estimate expression for each gene. The weights are inversely proportional to the variances. Our Model (2) to incorporate operon information is as follows.

For spotted array (SA) studies:
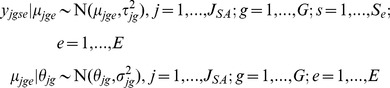



For oligonucleotide array studies:
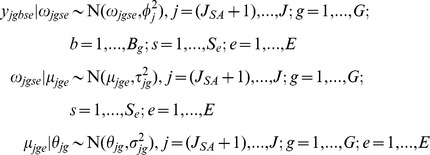



For all studies:
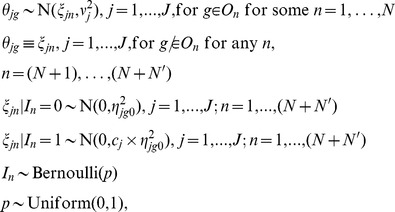
(2)


The values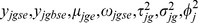 are as described above for Model (1). For *θ_jg_*, if gene *g* is a member of operon *O_n_*, the *θ_jg_* values are assumed to be normally distributed with the average expression equal to that of operon *n* in study *j*, with 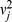 the study-specific operon variability. If gene *g* is not a member of any operon *O_n_*, *θ_jg_* is treated separately from other genes. Here, *n* ranges from 1 to the total number of operons *N* plus the number of genes not included in any operon *N′*. Similar to Model (1), Model (2) specifies each study separately, and does not combine mean expression levels for each study into an overall mean value. The normal assumption for log-expression ratios of genes organized in operons has been used by many previous authors, including Wang and Zhang [30], Price et al. [26], Xiao et al. [23], Iber [31], de Hoon et al. [32], Segal et al. [33]. In repeated microarray experiments, it is typical to model the log-expression ratios with a normal distribution. For genes organized in operons, the same bases for the model assumptions apply. We assume that genes within the same operon will have the same expression pattern for ratios between two conditions, on the log scale, for a steady-state condition. We assume that there will be some systematic error around the average log-expression ratio within an operon. Some genes will have log-ratios with higher values than the mean, and some will have lower, but the distribution will center with the highest probability at the mean, and lower probability for values much higher and lower. Thus, the log-ratios of expression for genes within an operon are assumed normally distributed.

We define *I_n_* ∼ Bernoulli(*p*) as the indicator variable for differential expression, i.e. ξ*_jn_* ≠ 0, *j* = 1,…, *J*, where *p* is the percent of differentially expressed genes. Thus, Prob(*I_n = _*1) = *p*, where
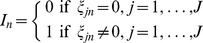



Here, genes are separated into two groups, non-expressed (*I_n = _*0) and expressed (*I_n = _*1) with probabilities (1-*p*) and *p*, respectively. When *I_n = _*0, the ξ*_jn_* are assumed to be normally distributed with mean zero and small variance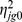; when *I_n = _*1, the ξ*_jn_* are assumed to be normally distributed with mean zero and large variance 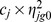. For each gene, Model (2) produces the posterior probability of differential expression, *D_g_* = Prob(*I_n = _*1 | data), which is the basis for inference.

### Prior Distributions for Models (1) and (2)

For prior distributions, we assign distributions that are as uninformative as possible which still result in convergence of the models. For parameters common to both Models (1) and (2), we assigned conjugate scaled inverse chi-squared prior distributions to the experiment, slide and probe variance parameters, 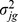, 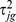, and 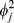, respectively. The scale parameters are derived from the data, by pooling information from all genes (similar to Tseng et al. [5]; Lönnstedt and Speed [8]; Gottardo et al. [10]; Conlon et al. [19], [20]). For Model (2), the prior distribution of operon variability 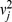 was assigned an inverse chi-squared distribution, with scale parameter equivalent to the variability within operons of each study. Note that we specify a common parameter for variance over all operons within each study (similar to Xiao et al. [23]). Further details on prior distributions are provided in Appendix S2: Prior distributions. The prior structure for Models (1) and (2) for individual studies is similar to that of Gottardo et al. [10], except that Models (1) and (2) generate posterior distributions for *p*, while Gottardo et al. calculate *p* using an iterative algorithm. Our data sets also have more levels of replication than the model of Gottardo et al., i.e. multiple probes, slides and experiments. The hierarchical structure of Models (1) and (2) for individual studies is also similar to the Bayesian ANOVA models (BAM) of Ishwaran and Rao [11], [12]. BAM redefines the identification of differentially expressed genes as a variable selection procedure, and employs a Bayesian model designed for adaptive shrinkage. Models (1) and (2) differ from BAM for individual studies, however, since BAM models are constructed for two-sample rather than one-sample data; Models (1) and (2) also have more levels of data replication. We produce posterior distributions for model parameters by implementing a Markov chain Monte Carlo (MCMC) procedure (details provided in Appendix S2). We calculate gene-specific posterior probabilities of differential expression for Models (1) and (2); the models are then compared using integration-driven discovery and Bayesian false discovery, defined in the following sections.

### Markov Chain Monte Carlo Procedure

We produce posterior distributions for model parameters by implementing a Markov chain Monte Carlo (MCMC) algorithm (details provided in Appendix S2). For the operon model, the estimated expression level of a gene is a weighted average of the gene-specific and operon-specific mean expression levels. The weights are inversely proportional to the variance values. We calculate gene-specific posterior probabilities of differential expression for Models (1) and (2); the models are then compared using integration-driven discovery and Bayesian false discovery, defined in the following sections. More detail on the MCMC implementation is provided in Appendix S2.

### Integration-driven Discovery

Choi et al. [16] introduced the integration-driven discovery rate (IDR) as the proportion of genes determined to be differentially expressed in meta-analysis but not in any of the individual studies alone. IDR depicts the gain in information from combining studies compared to individual analyses. We fix the threshold level of posterior probability of differential expression, γ, and label genes as differentially expressed if (*D_g_* ≥ γ). Specifically, IDR is defined as follows:
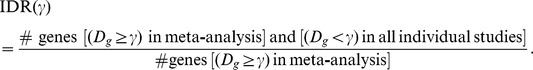



For the simulation data, true genes are defined as those that were simulated to be differentially expressed. The true integration-driven discovery rate, *t*IDR, is the proportion of true genes discovered in meta-analysis but not in any of the separate studies:
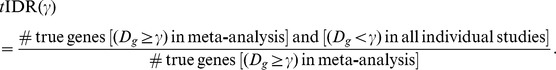



### Bayesian False Discovery Rate

The false discovery rate (FDR) was introduced by Benjamini and Hochberg [34] and is defined as the expected number of discoveries that are not truly differentially expressed divided by the total number of discoveries. Further analyses and discussions of FDR for microarray data are provided in Tusher et al. [35], Genovese and Wasserman [36], Storey [37] and Storey and Tibshirani [38]. For Bayesian analyses, Genovese and Wasserman [39] introduced the posterior expected FDR (*pe*FDR) as:
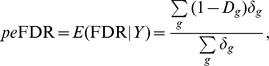
with δ_g_ an indicator variable for differentially expressed genes and ***Y*** representing the data (see also Do et al. [14]). Note that Conlon et al. [19] compared true FDR to *pe*FDR in several simulation studies and found that the two measures were always within 3% of each other on average. In addition, the *pe*FDR was a conservative estimate of true FDR in these simulation studies.

## Results

### Simulation Results for Two Studies

We simulated data for two studies similar to the biological data; Study 1 was specified to resemble the spotted array study, and Study 2 was similar to the ISO array study. We simulated a total of 3,000 genes and three values for the percent of differentially expressed genes: *p_s_* = 5%, 10%, 25% (*p_s_* denoting *simulated*); each slide was also standardized to have mean zero and unit standard deviation (similar to Shen et al. [17]; Conlon et al. [19], [20]). We simulated the operon structure similar to the predicted operon structure of the biological data. For genes within the same operon, we assumed a common average gene expression level, with variance again corresponding to the biological data. Appendix S1 provides further details on the simulation procedure.

**Figure 1:**
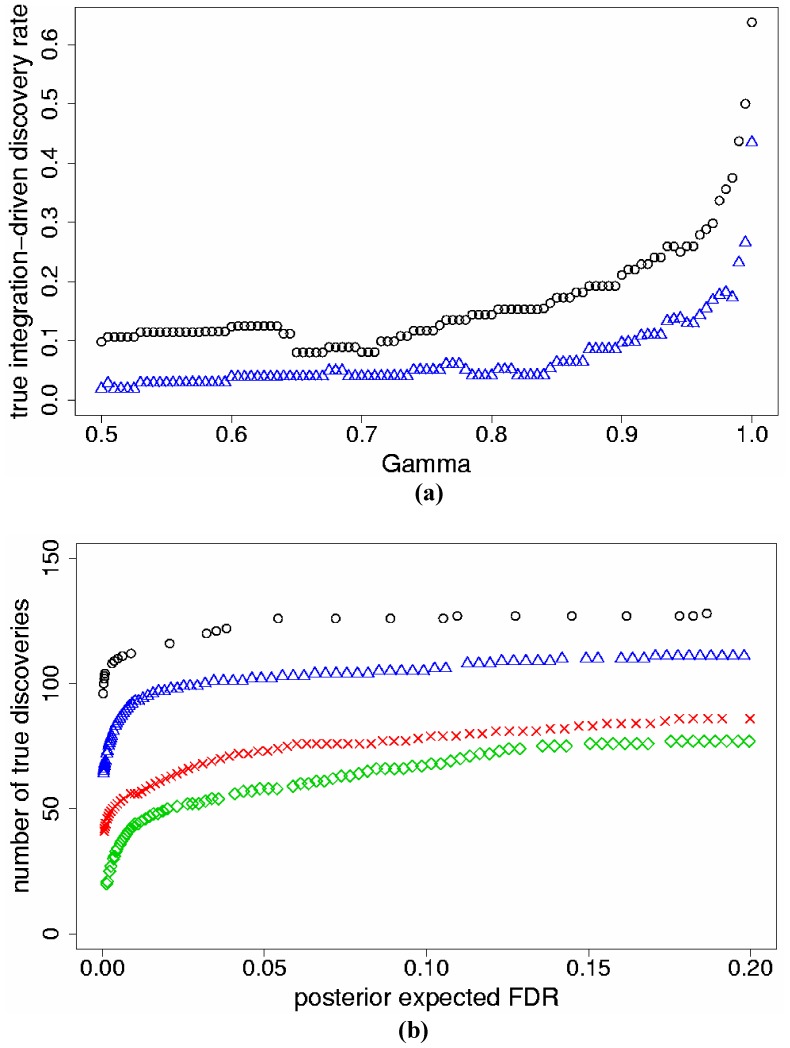
Results for the two-study simulation data with simulated percent differentially expressed genes *p_s_* = 5%. a) True integration-driven discovery rate (*t*IDR) versus levels of posterior probability of differential expression γ ≥0.50, for Model (1) (triangles) and Model (2) (circles); b) The maximum number of true genes discovered versus posterior expected false discovery rate (*pe*FDR) for Model (1) (triangles), Model (2) (circles), individual analyses of Study 1 (checks), Study 2 (diamonds).

We implemented Models (1) and (2) for the meta-analysis of two studies; each study was also analyzed separately using *j* = 1. Results are discussed here for the data set with *p_s_* = 5%. To compare Models (1) and (2), we calculated for both models the true integration-driven discovery rate (*t*IDR) for fixed levels of γ ≥0.50, which correspond to posterior probabilities of differential expression greater or equal to 50%. Model (2) produced higher *t*IDR than Model (1) for all values of γ ≥0.50 (Figure 1a). We also fixed threshold levels of *pe*FDR and found that Model (2) discovered more true genes than Model (1) for the same levels of *pe*FDR <20%; both models improved discoveries versus separate analyses (Figure 1b). Similar results for *t*IDR and *pe*FDR were determined for the data sets with *p_s_* = 10%, 25% (Table 1).

**Table 1:** Simulation results for two and five studies.

	*P_s_* = 5%		*P_s_* = 10%		*P_s_* = 25%	
	Model (1)	Model (2)	Model (1)	Model (2)	Model (1)	Model (2)
Two-Study Simulation Data						
*t*IDR, γ = 0.95	12.9%	25.9%	6.5%	21.9%	2.6%	15.3%
True Genes, *pe*FDR = 0.05	102	122	207	245	544	614
True Genes, Fixed Top *p_s_*%	111	127	232	259	609	657
Five-Study Simulation Data						
*t*IDR, γ = 0.95	5.3%	9.9%	1.9%	6.1%	1.6%	4.2%
True Genes, *pe*FDR = 0.05	141	150	277	293	719	735
True Genes, Fixed Top *p_s_*%	142	149	278	294	714	735

True integration-driven discovery rate (*t*IDR) for posterior probability of differential expression γ = 0.95, the number of true genes discovered for posterior expected false discovery rate *pe*FDR = 5%, and the number of true genes discovered for a fixed top number of genes. Results are shown for Models (1) and (2), and for the three values of simulated percent differentially expressed genes *p_s_*.

In addition to *t*IDR and *pe*FDR, researchers are often interested in the top set of genes only, e.g. the top 100 genes. For this reason, we ranked the genes based on *D_g_* in both Models (1) and (2) and compared the resulting numbers of true genes included in the top set of genes. Here, we chose a threshold of the top *p_s_*% of genes. We found that Model (2) identified more true genes than Model (1), for all data sets (Table 1).

### Simulation Results for Five Studies

We also implemented Models (1) and (2) to combine five independent studies. For this, we produced three additional simulation studies: one with a design similar to Study 1, and two with designs similar to Study 2. The simulation parameters were either within the range of the biological data, or somewhat outside the range; Appendix S1 provides further details on the simulation procedure. We again simulated three levels for the percent of differentially expressed genes: *p_s_* = 5%, 10%, 25%.

**Figure 2:**
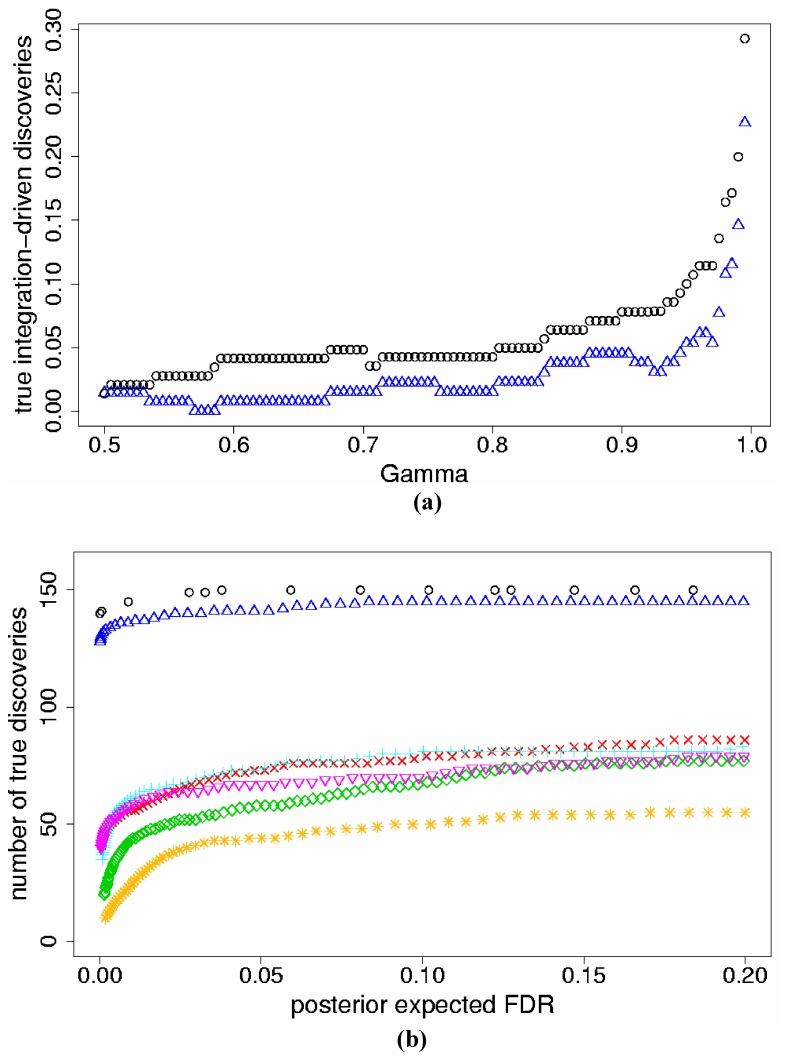
Results for the five-study simulation data with simulated percent differentially expressed genes *p_s_* = 5%. a) True integration-driven discovery rate (*t*IDR) versus levels of posterior probability of differential expression γ ≥0.50, for Model (1) (triangles) and Model (2) (circles); b) The maximum number of true genes discovered versus posterior expected false discovery rate (*pe*FDR) for Model (1) (triangles), Model (2) (circles), individual analyses of Study 1 (checks), Study 2 (diamonds), Study 3 (pluses), Study 4 (inverted triangles), Study 5 (stars).

For the data set corresponding to *p_s_* = 5%, Model (2) again identified higher *t*IDR than Model (1) for all levels of γ ≥0.50 (Figure 2a). In comparison to the two-study simulations, integrating more studies resulted in lower average *t*IDR for γ ≥50% for both Models (1) and (2). This occurred since, for larger numbers of studies, it was more likely that some genes had *D_g_* ≥ γ in at least one individual study, which reduced *t*IDR. Similar results were established for the data sets with *p_s_* = 10%, 25% (Table 1).

When combining five studies, both Models (1) and (2) identified more true discoveries than separate analyses for the same thresholds of *pe*FDR; Model (2) again discovered more true genes than Model (1), similar to the two-study findings (Figure 2b). In comparison to the two-study simulations, pooling more studies produced more true discoveries for the same levels of *pe*FDR, for both models. This indicates that combining more data improves the accuracy of *pe*FDR. When examining the top 150 genes (i.e. the top *p_s_*%), Model (2) again identified more true genes than Model (1), and pooling more studies improved the results versus the two study simulations. We found similar results for *pe*FDR and the top sets of genes for *p_s_* = 10%, 25% (Table 1).

### Biological Data Results

**Figure 3:**
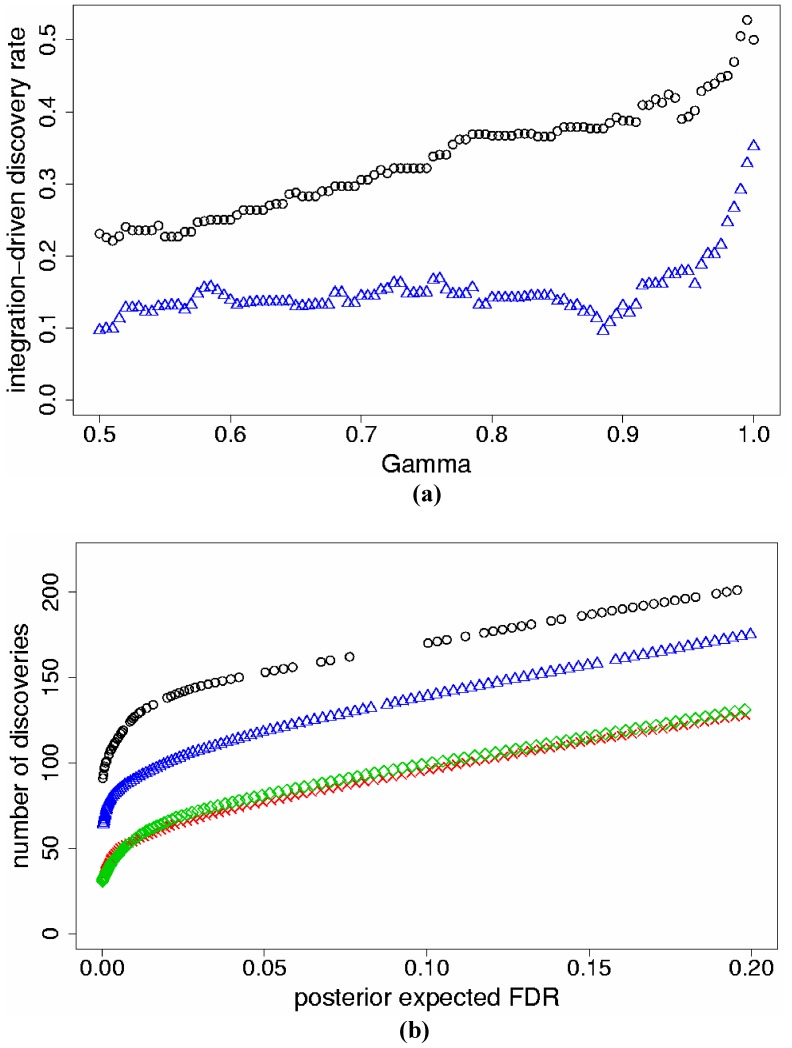
*G. sulfurreducens* spotted array and ISO array study data results. a) Integration-driven discovery rate (IDR) versus levels of posterior probability of differential expression γ ≥0.50, for Model (1) (triangles) and Model (2) (circles); b) The maximum number of genes discovered versus posterior expected false discovery rate (*pe*FDR) for Model (1) (triangles), Model (2) (circles), separate analyses of the *G. sulfurreducens* spotted array study (checks) and ISO array study (diamonds).

We implemented Models (1) and (2) to combine the nitrogen fixation data of *G. sulfurreducens* for the spotted array and ISO array studies; we also analyzed each study separately. In total, there were 3,323 genes that had expression in both studies (for further details of the biological data, see Appendix S1: Biological data). For IDR, our results were similar to the simulations studies; Model (2) produced higher IDR than Model (1) for all levels of γ ≥0.50 (Figure 3a). For fixed values of *pe*FDR <20%, both Models (1) and (2) discovered more genes than the individual studies alone, and Model (2) discovered more genes than Model (1) for all values (Figure 3b).

## Discussion

Here, we developed a new Bayesian meta-analysis model that incorporates operon information into the model. By borrowing information across genes in the same operon, we improved results versus previous Bayesian meta-analysis models that assume expression of a gene is independent of other genes. In simulations of two and five studies, we found that the operon model outperformed the independence model using three common comparison measures: the percent of true genes discovered in meta-analysis but not in separate studies, the number of true genes identified for the same thresholds of Bayesian false discovery, and the number of true genes discovered for a fixed top number of genes. For the biological data of *G. sulfurreducens*, the operon model produced higher integration-driven discovery rates for the same thresholds of posterior probability of differential expression than the independence model. The operon model also discovered more genes than the independence model for fixed levels of Bayesian false discovery. We note that Xiao et al. [23] introduced a Bayesian model for one study that incorporates operon information into the model. The operon model was shown to improve gene expression estimates compared to the independence model for one study. Here, we extended this model for multiple studies, showing similar improvement for the meta-analysis framework.

Our Bayesian meta-analysis operon model used the assumption that genes in an operon are co-transcribed. There are some cases where genes from an operon are expressed at different levels. First, genes may express differently due to their location in the operon. However, as discussed in Price et al. [26], in steady state cases, these differences do not affect the ratios of expression between the two experimental conditions; thus, expression ratios should be similar across an operon. Second, small noncoding RNAs can bind to specific transcripts and cause them to increase or decrease stability. However, in practical terms, genes in the same operon typically show similar patterns of expression, and patterns of expression are used to predict genes in the same operon (see also Sabatti et al. [24]; Price et al. [26]).

## Supporting Information

Appendix S1 Description of simulation data sets and biological data sets.
(DOC)

Appendix S2 Details of the Markov chain Monte Carlo implementation.
(DOC)
