# Supplementary material for: A Bayesian Model for Pooling Gene Expression Studies That Incorporates Co-Regulation Information
Source: PLoS One. 2012 Dec 28;7(12):e52137. doi: 10.1371/journal.pone.0052137 (PMC3532429; doi:10.1371/journal.pone.0052137)
Supplement: Appendix S1 — Description of simulation data sets and biological data sets. (DOC) [file pone.0052137.s001.doc]

**Appendix S1**

**Two study simulation data**

We simulated data for two studies, with a format designed to resemble the *G. sulfurreducens* spotted array and ISO array studies (details of the biological data are provided in Appendix A: Biological data). We simulated the percent of differentially expressed genes *p_s_* = 5%, 10%, 25% (*p_s_* denoting *simulated*), with 3,000 genes in each study. Study 1 was similar to the spotted array data, with 3 repeated experiments, each with 8 replicate arrays. Study 2 was similar to the ISO array study, with 3 replicate experiments, each with 2 repeated slides and 4 probes per gene on each array. Data was simulated from Model (2), with model parameters chosen to correspond to the biological data. For Study 1, we assigned $\eta_{jg0}^{2}=0.02$ and *c_j_* = 60. We set the variance across slides to 0.02, and across experiments to 0.03. For Study 2, we assigned $\eta_{jg0}^{2}=0.025$ and *c_j_* = 48. We set probe variance to 0.03, slide variance to 0.005, and experiment variance to 0.008. For the biological data, 75.1% of genes are predicted to be in operons, with an average operon size of 3.53. Note that the operon structure for the biological data is based on predictions, and many operons have not been experimentally verified. We used similar parameters for the simulation data, with 75% of genes in operons, and four genes per operon. We assigned operon variability of 0.073 for Study 1 and 0.039 for Study 2, similar to the biological data; we used common operon variance for all operons within a study, similar to other authors (e.g. Xiao et al. [1]). The biological data had inter-study variance of 0.02 for all 3,323 genes, and 0.12 for the top 5% of genes. Inter-study variability for the simulation data was similar to the biological data for all genes, and was higher than the biological data for the top genes, as follows. The simulated inter-study variance for all 3,000 genes was 0.03 for *p_s_* = 5%, 0.03 for *p_s_* = 10% and 0.04 for *p_s_* = 25%; and was 0.18 for the top percent of genes for *p_s_* = 5%, 0.14 for *p_s_* = 10% and 0.14 for *p_s_* = 25%. Each slide was standardized to have zero mean and unit standard deviation.

**Simulation data for five studies**

For the five study simulation data, Study 1 and Study 2 were the same as in the previous section, with data simulated for 3 additional studies. Study 3 was designed with format similar to Study 1, and Studies 4 and 5 had format similar to Study 2. The percent of differentially expressed genes was again *p_s_* = 5%, 10%, 25%, with 3,000 total genes. For $\eta_{jg0}^{2},$ *c_j_*, and probe, slide and experiment variance parameter values, we specified values that were either within the range of parameter values for the biological data, or somewhat outside this range. For Study 3, we assigned $\eta_{jg0}^{2}=0.025$ and *c_j_* = 50; the slide variance was set to 0.01 and experiment variance to 0.004. For Study 4, we set $\eta_{jg0}^{2}=0.02$ and *c_j_* = 55; the probe variance was specified to 0.04, slide variance to 0.004 and experiment variance to 0.009. For Study 5, we set $\eta_{jg0}^{2}=0.015$ and *c_j_* = 66.7; the probe variance was specified to 0.025, slide variance to 0.006 and experiment variance to 0.004. Inter-study variability for the five-study simulation data was somewhat larger than for the biological data. Inter-study variance for all 3,000 genes was 0.06 for *p_s_* = 5%, 0.06 for *p_s_* = 10% and 0.08 for *p_s_* = 25%; and 0.32 for *p_s_* = 5%, 0.30 for *p_s_* = 10% and 0.33 for *p_s_* = 25% for the top *p_s_*% of data. Each array was again standardized to have zero mean and unit standard deviation.

**Biological data**

*Geobacter* is a microbe that conducts electricity from organic waste matter to an electrode and gains energy by respiring iron oxides. Recently, two gene expression studies were conducted for *G. sulfurreducens* to identify genes in the nitrogen fixation pathway (Methé et al. [2]; Postier et al. [3]). Each study used a different microarray technology; the first consisted of spotted DNA arrays and the second used CombiMatrix *in-situ* synthesized oligonucleotide arrays. Each was a comparative study for two conditions: cells grown with ammonium versus cells that required atmospheric nitrogen fixation (referred to as the *treatment* condition); we provide further details in the following.

***Spotted DNA microarrays***

The spotted DNA amplicon microarrays consisted of 3,417 spots which characterized the predicted coding sequences of *G. sulfurreducens*. For the study of nitrogen fixation, three independent repeated experiments were conducted, each producing six arrays, for a total of eighteen. The arrays were processed using the TIGR Spotfinder software (Saeed et al. [4]), and the Lowess procedure was used to normalize the intensity values (for further details, please see Methé et al. [2]). We used the post-normalized log_2_-expression ratios, and standardized each slide to have mean zero and unit standard deviation. The microarray data was deposited into the ArrayExpress Database, with corresponding Accession Numbers E-TIGR-81, E-TIGR-82, and A-TIGR-17 (Brazma et al. [5]).

*Combimatrix* in-situ *synthesized oligonucleotide arrays*

Electrochemical *in-situ* oligonucleotide synthesis technology is used to produce CombiMatrix microarrays. The oligonucleotides are created on the silicon chip using electrochemical control; individual oligomers are of width 35-40 bases (Liu et al. [6,7]). The *G. sulfurreducens* arrays and oligomers were developed using the NCBI genome sequence, Accession Number AE017180.1 (Methé et al. [8]), which consisted of 3,447 genes. Each microarray consisted of 12,000 features, with each gene specified by up to four probes. For the study of nitrogen fixation, three repeated independent experiments were carried out, each with two technical replicates. The results were analyzed using GenePix and Acuity version 4.0 software (Axon Instruments, Union City, California, United States), and the Lowess procedure was used to normalize the intensity values (further details provided in Postier et al. [3]). We used the post-normalized log_2_-expression ratios, and again standardized each array to have zero mean and unit standard deviation. The microarray data was deposited in the NCBI Gene Expression Omnibus Database, with corresponding Accession Number GSE7147 (Edgar et al. [9]).

**References**

1. Xiao G, Martinez-Vaz B, Pan W, Khodursky AB (2006) Operon information improves gene expression estimation for cDNA microarrays. BMC Genomics 7: 87.
2. Methé BA, Webster J, Nevin K, Butler J, Lovley DR (2005) DNA microarray analysis of nitrogen fixation and Fe(III) reduction in Geobacter sulfurreducens. Appl Environ Microbiol 71: 2530-2538.
3. Postier B, DiDonato R, Nevin K, Liu A, Frank B, et al. (2008) Benefits of in-situ synthesized microarrays for analysis of gene expression in understudied microorganisms. J Microbiol Methods 74: 26-32.
4. Saeed AI, Sharov V, White J, Li J, Liang W, et al. (2003) TM4: a free, open-source system for microarray data management and analysis. Biotechniques 34: 374-378.
5. Brazma A, Parkinson H, Sarkans U, Shojatalab M, Vilo J, et al. (2003) ArrayExpress - a public repository for microarray gene expression data at the EBI. Nucleic Acids Res 31: 68-71.
6. Liu RH, Dill K, Fuji HS, McShea A (2006) Integrated microfluidic biochips for DNA microarray analysis. Expert Rev Mol Diagn 6: 253-261.
7. Liu RH, Nguyen T, Schwarzkopf K, Fuji HS, Petrova A, et al. (2006) Fully integrated miniature device for automated gene expression DNA microarray processing. Anal Chem 78: 1980-1986.
8. Methé BA, Nelson KE, Eisen JA, Paulsen IT, Nelson W, et al. (2003) Genome of Geobacter sulfurreducens: metal reduction in subsurface environments. Science 302: 1967-1969.
9. Edgar R, Domrachev M, Lash AE (2002) Gene Expression Omnibus: NCBI gene expression and hybridization array data repository. Nucleic Acids Res 30: 207-210.
