# Supplementary material for: A Bayesian Model for Pooling Gene Expression Studies That Incorporates Co-Regulation Information
Source: PLoS One. 2012 Dec 28;7(12):e52137. doi: 10.1371/journal.pone.0052137 (PMC3532429; doi:10.1371/journal.pone.0052137)
Supplement: Appendix S2 — Details of the Markov chain Monte Carlo implementation. (DOC) [file pone.0052137.s002.doc]

**Appendix S2**

**Markov chain Monte Carlo implementation**

We use a Markov chain Monte Carlo (MCMC) method to produce the posterior distributions of each of the parameters of Models (1) and (2). MCMC methods create samples from a density *p*(*κ*) for a parameter *κ* (where *p*(*κ*) may be known only up to a proportionality constant) by producing a Markov chain on the state space of *κ* with *p* as its true (stationary) distribution (for further details, please see Liu [1]). For Model (1), the joint posterior distribution is specified as follows:

$$\begin{matrix} p(y_{jgse},y_{jgbse},\mu_{jge},\omega_{jgse},\tau_{jg}^{2},\sigma_{jg}^{2},\varphi_{j}^{2},\theta_{jg},I_{g},p,\eta_{jg0}^{2},c_{j})\text{ }= \\ \left[\prod_{j=1}^{J_{SA}} \prod_{g=1}^{G} \prod_{e=1}^{E} \left\{\prod_{s=1}^{S_{e}} p(y_{jgse}|\text{ }\mu_{jge},\tau_{jg}^{2})p(\mu_{jge}|\text{ }\theta_{jg,}\sigma_{jg}^{2})\right\}\times\text{ }\right. \\ \prod_{j=(J_{SA}+1)}^{J} \prod_{g=1}^{G} \prod_{b=1}^{B_{g}} \prod_{e=1}^{E} \left\{\prod_{s=1}^{S_{e}} p(y_{jgbse}|\text{ }\omega_{jgse},\varphi_{j}^{2})p(\omega_{jgse}|\text{ }\mu_{jge},\tau_{jg}^{2})p(\mu_{jge}|\text{ }\theta_{jg,}\sigma_{jg}^{2})\right\}\times\\ \left.p\text{(}\theta_{jg},I_{g}|p,\Omega_{j})p(\varphi_{j}^{2})p(\tau_{jg}^{2})p(\sigma_{jg}^{2})p(p)p(\Omega_{j})\right] \end{matrix}$$

with $\Omega_{j}=(\eta_{jg0}^{2},c_{j})$, *j* = study, *g* = gene, *b* = probe, *e* = experiment, *s* = slide.

For Model (2), the joint posterior distribution is:

$$\begin{matrix} p(y_{jgse},y_{jgbse},\mu_{jge},\omega_{jgse},\tau_{jg}^{2},\sigma_{jg}^{2},\varphi_{j}^{2},\theta_{jg},\xi_{jn},\nu_{j}^{2},I_{n},p,\eta_{jg0}^{2},c_{j})\text{ }= \\ \left[\prod_{j=1}^{J_{SA}} \prod_{g=1}^{G} \prod_{e=1}^{E} \left\{\prod_{s=1}^{S_{e}} p(y_{jgse}|\text{ }\mu_{jge},\tau_{jg}^{2})p(\mu_{jge}|\text{ }\theta_{jg,}\sigma_{jg}^{2})\right\}\times\text{ }\right. \\ \prod_{j=(J_{SA}+1)}^{J} \prod_{g=1}^{G} \prod_{b=1}^{B_{g}} \prod_{e=1}^{E} \left\{\prod_{s=1}^{S_{e}} p(y_{jgbse}|\text{ }\omega_{jgse},\varphi_{j}^{2})p(\omega_{jgse}|\text{ }\mu_{jge},\tau_{jg}^{2})p(\mu_{jge}|\text{ }\theta_{jg,}\sigma_{jg}^{2})\right\}\times\\ \left.p(\theta_{jg}|\xi_{jn},\nu_{j}^{2})p\text{(}\xi_{jn},I_{n}|p,\Omega_{j})p(\varphi_{j}^{2})p(\tau_{jg}^{2})p(\sigma_{jg}^{2})p(\nu_{j}^{2})p(p)p(\Omega_{j})\right] \end{matrix}$$

where $\Omega_{j}=(\eta_{jg0}^{2},c_{j})$, *j* = study, *g* = gene, *b* = probe, *e* = experiment, *s* = slide, *n* = index for operon number or individual gene number; *n* ranges from 1 to the total number of operons *N* plus the number of genes not included in any operon *N’*.

**Prior distributions**

***Prior distributions for parameters common to Models (1) and (2)***

For the prior distributions, we use some information from the data, but we assign as uninformative priors as possible which result in model convergence. For parameter *p*, we specified an uninformative Uniform(0,1) distribution. The prior distributions of the parameters for probe, slide and experiment effect variance, $\varphi_{j}^{2}$, $\tau_{jg}^{2}$ and $\sigma_{jg}^{2}$, respectively, require some information from the data. We specified the following conjugate scaled inverse chi-squared prior distributions for these values (see also Tseng et al. [2]; Lönnstedt and Speed [3]); Gottardo et al. [4]; Conlon et al. [5],[6]; Xiao et al. [7]):

$$\begin{matrix} \varphi_{j}^{2}\text{ } \sim\text{ }h\tilde{\varphi}_{j}^{2}/\chi_{h}^{2} \\ \tau_{jg}^{2}\text{ } \sim\text{ }k\tilde{\tau}_{j}^{2}/\chi_{k}^{2} \\ \sigma_{jg}^{2}\text{ } \sim\text{ }m\tilde{\sigma}_{j}^{2}/\chi_{m}^{2} \end{matrix}$$

In these specifications, $\tilde{\varphi}_{j}^{2}$, $\tilde{\tau}_{j}^{2}$ and $\tilde{\sigma}_{j}^{2}$ are scale parameters for the inverse chi-squared distribution calculated by combining information from all genes. For the spotted array studies, $\tilde{\tau}_{j}^{2}$and$\tilde{\sigma}_{j}^{2}$ are obtained as follows:

$$\tilde{\tau}_{j}^{2}\text{ }=\text{ }\frac{1}{G\left(\sum S_{e}-1\right)}\sum_{g=1}^{G} \sum_{e=1}^{E} \sum_{s=1}^{S_{e}} \left(y_{jgse}-y_{jg\cdot e}\right)^{2},$$

where *y_jg.e_* is the mean log-ratio of expression over arrays within an experiment:

$$y_{jg\cdot e}\text{ }=\text{ }\frac{1}{S_{e}}\sum_{s=1}^{S_{e}} y_{jgse}$$

Similarly, the scale parameter for $\sigma_{jg}^{2}$ is computed as follows:

$$\tilde{\sigma}_{j}^{2}=\frac{1}{G(E-1)}\sum_{g=1}^{G} \sum_{e=1}^{E} (y_{jg\cdot e}-y_{jg\cdot\cdot})^{2},$$

where *y_jg.._* is the mean log-expression ratio over both slides and experiments.

For ISO array studies, the scale parameters for $\varphi_{j}^{2}$, $\tau_{jg}^{2}$and $\sigma_{jg}^{2}$ are derived by the following:

$$\tilde{\varphi}_{j}^{2}\text{ }=\text{ }\frac{1}{G\left(\sum S_{e}\right)\left(R_{g}-1\right)}\sum_{g=1}^{G} \sum_{e=1}^{E} \sum_{s=1}^{S_{e}} \sum_{r=1}^{R_{g}} \left(y_{jgrse}-y_{jg\cdot se}\right)^{2},$$

where *y_jg.se_* is the mean log-ratio of expression over probes within an array:

$$y_{jg\cdot se}\text{ }=\text{ }\frac{1}{B_{g}}\sum_{b=1}^{B_{g}} y_{jgbse}$$

Here, the scale parameter for the slide variance is obtained as follows:

$$\tilde{\tau}_{j}^{2}\text{ }=\text{ }\frac{1}{G\left(\sum S_{e}-1\right)}\sum_{g=1}^{G} \sum_{e=1}^{E} \sum_{s=1}^{S_{e}} \left(y_{jg\cdot se}-y_{jg\cdot\cdot e}\right)^{2},$$

where *y_jg..e_* is the mean log-ratio of expression over probes and arrays within an experiment:

$$y_{jg\cdot\cdot e}\text{ }=\text{ }\frac{1}{S_{e}\left(B_{g}\right)}\sum_{s=1}^{S_{e}} \sum_{b=1}^{B_{g}} y_{jgbse}$$

Similarly, the scale parameter for $\sigma_{jg}^{2}$ is computed by the following:

$$\tilde{\sigma}_{j}^{2}=\frac{1}{G(E-1)}\sum_{g=1}^{G} \sum_{e=1}^{E} (y_{jg\cdot\cdot e}-y_{jg\cdot\cdot\cdot})^{2},$$

where *y_jg..._* is the mean log-expression ratio over probes, slides and experiments.

We assigned three degrees of freedom in each study for $\varphi_{j}^{2}$, $\tau_{jg}^{2}$and $\sigma_{jg}^{2}$, i.e. *h* = *l* = *m* = 3, for distributions that are as uninformative as possible. The following distributions were specified for the remaining values.

$$\begin{matrix} \eta_{jg0}^{2} \text{ }\sim\text{ }as_{1}^{2}/\chi_{a}^{2} \\ c_{j} \text{ }\sim\text{ }bs_{2}^{2}/\chi_{b}^{2} \end{matrix}$$

The degrees of freedom and scale parameters *a*, $s_{1}^{2}$, respectively, were assigned so that the prior mean of $\eta_{jg0}^{2}$ was 1 with variance 1, and *b*, $s_{2}^{2}$ were specified so that the prior mean of *c_j_* was 100 with variance 10,000. These parameter specifications are suitable for microarrays that have been standardized to have zero mean and unit standard deviation (for further detail on prior assignments, please see Conlon et al. [5]).

***Prior distributions for parameters specific to Model (2) only***

For the prior distribution of operon variance, $\nu_{j}^{2}$, we assigned an inverse chi-squared distribution, with scale parameter equivalent to the mean study-specific variability within operons of the data. We again assigned 3 degrees of freedom, so that the distribution was as uninformative as possible. Note that $\nu_{j}^{2}$ is a common parameter for operon variance for all operons within a study (similar to Xiao et al. [7]).

**Full conditional posterior distributions**

We sampled each parameter from the full conditional posterior distributions by implementing an MCMC procedure using the WinBUGS software (Spiegelhalter et al. [8]). For all posterior inference, we used 5,000 iterations after convergence, similar to other authors (Tseng et al. [2]; Conlon et al. [5],[6]), which was more than adequate. Each parameter was verified to achieve convergence using standard Bayesian model convergence diagnostics, including history plots, density plots, and autocorrelation statistics. Further details on the MCMC procedure can be found in Conlon et al. [5].

**Number of model parameters**

The number of parameters estimated by the Bayesian models is specific to each gene. For the independence Model (1), we estimate a total of 22 parameters for each gene, plus 3 parameters that are common to all genes. There are 42 data points measured for each gene for the biological data. For Model (2) to incorporate operon information, we add two model parameters for each gene, for a total of 24 parameters estimated for each gene; we also add two additional parameters that are estimated for all genes simultaneously. The number of model parameters for the simulation data for two studies is similar to the biological studies. For the simulation data for five studies, there are 57 parameters estimated for each individual gene for Model (1), and 6 parameters that are estimated for all genes simultaneously. For Model (2) for the simulation data for five studies, there are 62 parameters estimated for each individual gene, and 11 parameters that are estimated for all genes simultaneously. The simulation data for five studies contains 120 data points for each gene.

**References**

1. Liu JS (2001) Monte Carlo Strategies in Scientific Computing. New York: Springer-Verlag.
2. Tseng GC, Oh MK, Rohlin L, Liao JC, Wong WH (2001) Issues in cDNA microarray analysis: quality filtering, channel normalization, models of variations and assessment of gene effects. Nucleic Acids Res 29: 2549-2557.
3. Lönnstedt I, Speed, TP (2002) Replicated microarray data. Stat Sin 12: 31–46.
4. Gottardo R, Pannucci JA, Kuske CR, Brettin T (2003) Statistical analysis of microarray data: a Bayesian approach. Biostatistics 4: 597-620.
5. Conlon EM, Song JJ, Liu JS (2006) Bayesian models for pooling microarray studies with multiple sources of replications. BMC Bioinformatics 7: 247.
6. Conlon EM, Song JJ, Liu A (2007) Bayesian meta-analysis models for microarray data: a comparative study. BMC Bioinformatics 8: 80.
7. Xiao G, Martinez-Vaz B, Pan W, Khodursky AB (2006) Operon information improves gene expression estimation for cDNA microarrays. BMC Genomics 7: 87.
8. Spiegelhalter DJ, Thomas A, Best NG (2003) WinBUGS Version 1.4 User Manual: MRC Biostatistics Unit.
